# Quantitative analysis of the relationship between the myocardial bridge and the FAI of pericoronal fat on computed tomography

**DOI:** 10.1038/s41598-024-55005-9

**Published:** 2024-03-12

**Authors:** Dan Zhang, Xin Tian, Meng-Ya Li, Hao-Wen Zhang, Yang Yu, Tong Pan, Cai-Ying Li

**Affiliations:** 1 Department of Medical Imaging, The Second Hospital of Hebei Medical University, 215 West Heping Road, Shijiazhuang, 050011 Hebei China; 2 Department of Radiology, The Third Hospital of Hebei Medical University, 139 Ziqiang Road, Shijiazhuang, 050000 Hebei China; 3 Department of Medical Imaging, Hebei General Hospital, Shijiazhuang, 050000 Hebei China

**Keywords:** Myocardial bridging (MB), Fat attenuation index (FAI), Coronary computed tomography angiography (CCTA), Epicardial adipose tissue (PCAT), Cardiovascular biology, Tomography

## Abstract

We performed this cohort study to investigate whether the myocardial bridge (MB) affects the fat attenuation index (FAI) and to determine the optimal cardiac phase to measure the volume and the FAI of pericoronary adipose tissue (PCAT). The data of 300 patients who were diagnosed with MB of the left anterior descending (LAD) coronary artery were retrospectively analyzed. All of patients were divided into the MB group and the MB with atherosclerosis group. In addition, 104 patients with negative CCTA results were enrolled as the control group. There was no significant difference between FAI values measured in systole and diastole (*P* > 0.05). There was no significant difference in FAI among the MB group, the MB with atherosclerosis group, and the control group (*P* > 0.05). In MB with atherosclerosis group, LAD stenosis degree (< 50%) (OR = 0.186, 95% CI 0.036–0.960; *P* = 0.045) and MB located in the distal part of LAD opening (OR = 0.880, 95% CI 0.789–0.980; *P* = 0.020) were protective factors of FAI value. A distance (from the LAD opening to the proximal point of the MB) of 29.85 mm had the highest predictive value for abnormal FAI [area under the curve (AUC), 0.798], with a sensitivity of 81.1% and a specificity of 74.6%.

## Introduction

Coronary artery myocardial bridge (MB) is a congenital coronary artery abnormality^1^. The coronary artery and its branches are usually located in the epicardial adipose tissue. When one or part of the coronary artery tunnels through the myocardium, it is called “myocardial bridge”^2^. MB can occur in any coronary artery branch, but the common site is the proximal and middle segments of the left anterior descending branch (67–98%)^3^, which is more likely to be symptomatic.

Myocardial bridges affect the morphology and function of coronary arteries^4^, and change microcirculation in pericoronary fat, leading to inflammation^5^ and promoting the formation of atherosclerotic plaque in proximal segments of coronary arteries, even myocardial infarction^6^.

The fat attenuation index (FAI) of pericoronary adipose tissue (PCAT) is defined as the mean CT value of PCAT within the radial distance from the outer wall of the coronary artery equal to the diameter of the coronary artery. FAI is a novel imaging biomarker of coronary artery inflammation^7^ and can be measured on cardiac CT images.

At present, there is a lack of research on the relationship between myocardial bridge and FAI, and there is no research on the measurement of FAI value in cardiac systole or diastole. We employed an artificial intelligence software for quantitative assessment of the FAI. The computational principle of the software is based on CCTA images, using segmentation algorithms. According to the literature of The Lancet^8^, the software extends 4 mm outward from the vessel wall and uses a threshold of − 190 HU to − 30 HU for the pericoronary fat segmentation. Through the 3D rendering module, the quantitative information of the pericoronary fat is color-coded and mapped onto the MPR original image, CPR, probe images, and CPR straightening graph. This study aimed to quantitatively evaluate the FAI in patients with myocardial bridge and the relationship between FAI and the anatomical characteristics of MB, and to explore the factors influencing FAI measurement.

## Method

### Subjects

**Figure 1:**
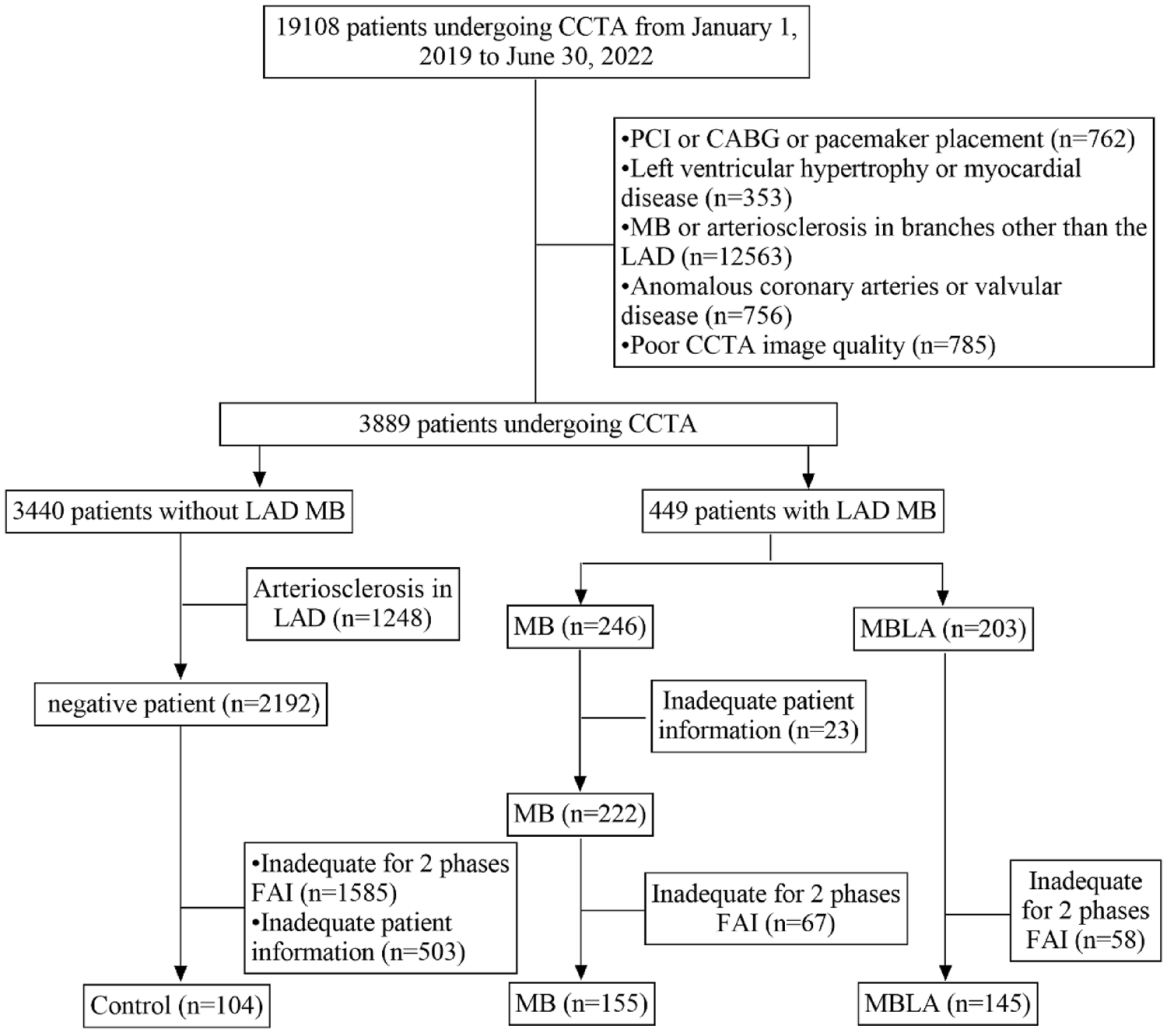
Flow chart of the study. *CCTA* coronary computed tomography angiography, *LAD* left anterior descending artery, *MB* myocardial bridging, *MBLA* myocardial bridging and LAD atherosclerosis, *FAI* fat attenuation index.

A total of 300 patients with only myocardial bridge of LAD were retrospectively enrolled, who received coronary computed tomography angiography (CCTA) examination in our hospital from January 2019 to June 2022. The flow chart is shown in Fig. 1.

Inclusion criteria:

1. CCTA confirmed MB in left anterior descending artery;
2. CCTA confirmed left anterior descending coronary atherosclerosis;
3. CCTA confirmed that there was no myocardial bridge and coronary atherosclerosis in other coronary artery branches except the left anterior descending artery;
4. There were no artifacts in systolic and diastolic images.

Exclusion criteria:

1. Cardiomyopathy and valvular disease;
2. Abnormal origin and termination of coronary artery, coronary aneurysm;
3. The CCTA image quality was poor;
4. Patients after coronary intervention and coronary artery bypass grafting;
5. Cardiac pacemaker, occluder, artificial valve and other cardiac implants;
6. CCTA confirmed myocardial bridge of other branches of coronary artery;
7. CCTA confirmed coronary arteriosclerosis of other branches of coronary artery.

In addition, 104 patients who underwent a CCTA examination for chest tightness or a routine physical examination with negative CCTA results were enrolled as the control group. The flow chart is shown in Fig. 1.

Inclusion criteria:

1. CTA result was negative;
2. CCTA confirmed that there was no myocardial bridge in any coronary artery;
3. There was no artifacts in systolic and diastolic images.

Exclusion criteria:

1. CCTA confirmed that the origin and termination of coronary artery were abnormal;
2. The CCTA image quality was poor.

Using a threshold of − 70.1^8^, patients in both the MB group and the MB with atherosclerosis group were categorized into two groups: the normal FAI group (FAI < − 70.1) and the abnormal FAI group (FAI ≥ − 70.1). Subsequently, an analysis of the factors influencing FAI was conducted.

According to whether contained calcification, plaques were identified as three types: calcified, non-calcified, and mixed plaques.

The degree of coronary stenosis was classified as ≥ 50% and < 50%.

### Ethics statement

This study was approved by the Ethics Committee of the Second Hospital of Hebei Medical University. The informed consent was waived for this study by the Ethics Committee of the Second Hospital of Hebei Medical University. All methods were performed in accordance with the relevant guidelines and regulations.

### Cardiac CT acquisition

A Phillips 256-slice spiral CT was used. The patient was in a supine position during the scan. A single end-expiratory breath-holding scan with retrospective ECG gating technique was performed. The breath-holding time was 5–7 s, and the scanning area began from 0.5 cm below the bifurcation of the autonomous trachea to the diaphragm of the heart. The intelligent tracking method was used to set the threshold of aortic layer to 150 HU, and the formal scan was started at 6 s after reaching the threshold. The non-ionic contrast agent Iohexol (350 mgI/ml) was injected with a double-barrel high-pressure injector at a rate of 4–5 ml/s. The dose was 0.8 ml/kg. Scanning parameters were as follows: tube current 320 mAs/revolution, tube voltage 120 kV, detector collimation 128 × 0.625, pitch 0.18, matrix 512 × 512, rotation time 330 ms, scanning field of vision 250 mm. The images of systolic phase (45% cardiac phase) and the images of diastolic phase (75% cardiac phase) were reconstructed.

### Measurements

#### Measurement methods

Imported all the original images of CCTA into the post-processing workstation Phillips EBW 6.0, and used cross section, volume rendering (VR), multiplanar reconstruction (MPR) and surface reconstruction (CPR) to analyze the systolic and diastolic images. The anatomic parameters of myocardial bridge were measured by two radiologists with more than 5 years of CCTA related work experience, and each parameter was measured three times to get the average value.

**Figure 2:**
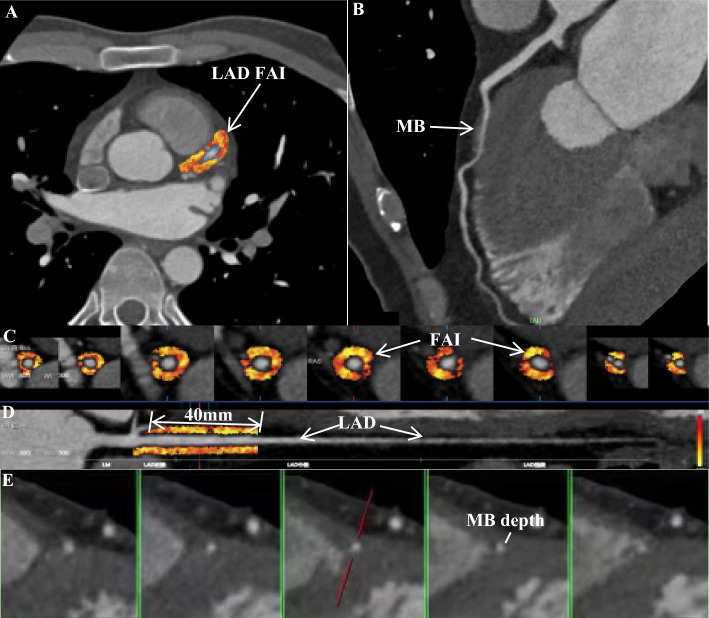
The method to quantify FAI and MB on cardiac computed tomography angiography images. (**A**) The axial plane image indicates the LAD and the FAI. (**B**) The axial plane image indicates the LAD and the MB. (**C**) Multiple axial plane images demonstrate the measurement of the FAI. (**D**) The FAI in the 40-mm proximal segment of the LAD is measured in a straightened view. (**E**) The axial plane image indicates the LAD and the MB depth. *FAI* fat attenuation index, *LAD* left anterior descending artery, *MB* myocardial bridging.

The length of the myocardial bridge and the position of the myocardial bridge (the distance from the opening of the anterior descending coronary artery to the proximal point of the myocardial bridge) were measured on the diastolic phase images. The depth of the myocardial bridge (the thickness of the myocardium covering the coronary artery during diastole) was measured on the cross-sectional images, as shown in Fig. 2. When the maximum myocardial thickness is ≤ 1 mm, it is uniformly recorded as 1 mm^9^.

The reference formula is as follows^10–12^:

$${\text{Systolic stenosis rate of myocardial bridge}} = \frac{{\text{Diastolic diameter}} - {\text{Systolic diameter}}}{{\text{Diastolic diameter}}} \times {100}$$

$${\text{Stenosis rate of MB}} = \frac{{\text{Diameter of coronary artery proximal to MB}} - {\text{Minimal diameter of MB}}}{{\text{Diameter of coronary artery proximal to MB}}} \times 100$$

$${\text{Muscle index}} = {\text{MB length}} \times {\text{MB depth}}$$

#### FAI and PCAT volume measurements

Based on an artificial intelligence software (Shukun, FAI, V1.7, Beijing, China), the 40-mm proximal segment of the LAD was traced and used to calculate FAI and PCAT volume. The software is based on various deep learning techniques, including CNN, and employs multiple deep learning composite networks to achieve fully automated vascular segmentation for a rapid and comprehensive diagnosis of all vessels. The cross-section images perpendicular to the vessel centerline were reconstructed, and the attenuation value of corresponding voxels around the artery is estimated as FAI calculation. The CT threshold of fat was − 190 to − 30 HU. PCAT was defined as adipose tissue within the distance from the outer wall of the vessel equal to the diameter of the vessel^12,13^. FAI was defined as the average CT attenuation value of PCAT. The FAI ≥ − 70.1 was abnormal^13^. A representative image of FAI analysis is shown in Fig. 2.

### Statistical analysis

SPSS software (version 25.0) was used for statistical analysis of the data. The measurement data conforming to the normal distribution was expressed by mean ± standard deviation. The comparison between the two groups was conducted by *t* test, and the comparison between multiple groups was conducted by one-way analysis of variance. The measurement data that did not conform to the normal distribution were expressed as median (IQR). The comparison between the two groups used Mann–Whitney *U* test, and the comparison between multiple groups used Kruskal–Wallis *H* test. If there was statistical difference, the comparison between the two groups would be carried out afterwards. The categorical variables were expressed as frequency or percentage, and the differences between groups were compared by chi-square test. Binary logistic regression analysis was used to screen the influencing factors of abnormal FAI. A *P* value < 0.05 was considered statistically significant. The ROC curve of myocardial bridge location was plotted, and the area under the curve (AUC) was calculated, and the best cutoff value was obtained by Youden index. Jorden index = sensitivity + specificity − 1, the maximum value of Jorden index, the corresponding index is the best critical point.

## Results

### Participants characteristics

A total of 155 patients were enrolled in the MB group (median age 53 (13) years, 82 men, 73 women), and 145 patients were in the MB with atherosclerosis group (median age 55 (10) years, 83 men, 62 women). There were 104 patients in the control group (median age 54 (14) years, 25 men, 79 women). Baseline characteristics of the MB group, the MB with atherosclerosis group, and the control group were compared. The results showed that the incidence rate and smoking rate of men in the MB group and MB with atherosclerosis group are higher than those in the control group (*P* < 0.05) (Table 1).

**Table 1:** Baseline characteristics of all participants.

Variables	Case group (n = 300)		*P*	Control (n = 104)	*P*
	MB (n = 155)	MBLA (n = 145)			
Patients					
Age (y)(median, interquartile range)	53.0 (13.0)	55.0 (10)	0.054	54.0 (14)	0.156
M (n, %)	82 (52.9)	83 (57.2)	0.450	25 (24)	< 0.001*
BMI (mean, range)	24.5 (22.9–26.1)	24.8 (23.0–26.7)	0.262	24.9 (22.6–27.6)	0.434
Risk factors					
Hypertension (n, %)	36 (23.2)	43 (29.7)	0.206	32 (30.8)	0.314
Hyperlipidemia (n, %)	16 (10.3)	26 (17.9)	0.058	16 (15.4)	0.161
Diabetes (n, %)	15 (9.7)	18 (12.4)	0.449	9 (8.7)	0.589
Smoking (n, %)	27 (17.4)	15 (10.3)	0.078	6 (5.8)	0.014*
Symptoms					
Angina pectoris (n, %)	13 (8.4)	18 (12.4)	0.252	4 (3.8)	0.366
Atypical angina (n, %)	10 (6.5)	8 (5.5)	0.733	5 (4.8)	0.849
Chest tightness and pain (n, %)	14 (9.0)	19 (13.1)	0.260	7 (6.7)	0.227
None (n, %)	118 (76.1)	100 (69.0)	0.164	88 (84.6)	0.017*
MB					
Length (mm)	22.0 (15.4–32.4)	22.8 (15.8–28.2)	0.467	–	–
Depth (mm)	1.5 (1.0–3.1)	1.0 (1.0–2.2)	0.025*	–	–
Location (mm)	34.8 (28.2–45.6)	35.9 (28.1–46.1)	0.659	–	–
MBMI	33.2 (18.5–81.1)	28.0 (18.4–54.9)	0.083	–	–
Systolic compression index	0.08 (0.05–0.15)	0.08 (0.05–0.13)	0.754	–	–
MB stenosis rate in diastole	0.32 ± 0.13	0.31 ± 0.13	0.814	–	–
MB stenosis rate in systole	0.32 (0.23–0.44)	0.32 (0.23–0.42)	0.832	–	–
In the diastole					
FAI	− 82.5 ± 6.9	− 83.3 ± 7.2	0.309	− 83.1 ± 8.1	0.592
PCAT	1845.7 ± 447.0	1893.0 (1585.5–2248.0)	0.270	2049.6 ± 459.3	0.001*
In the systole					
FAI	− 81.0 (− 86.0 to 76.0)	− 83.3 ± 8.5	0.072	− 82.8 ± 7.6	0.151
PCAT	1686.8 ± 431.9	1760.9 ± 466.8	0.085	1891.0 ± 476.6	0.001*
Plaques					
Calcification (n, %)	–	68 (37.0)	–	–	–
None-calcification (n, %)	–	97 (52.7)	–	–	–
Mixed (n, %)	–	19 (10.3)	–	–	–
Stenosis					
< 50% (n, %)	–	93 (64.1)	–	–	–
≥ 50% (n, %)	–	52 (35.9)	–	–	–

*MB* myocardial bridging, *MBLA* myocardial bridging and LAD atherosclerosis, *BMI* body mass index, *MBMI* MB muscle index, *LAD* left anterior descending coronary artery, *FAI* fat attenuation index, *PCAT* pericoronary adipose tissue. *P < 0.05.

For comparison of anatomical characteristics of MB among the groups, the results showed that the myocardial bridge depth in the MB group was greater than that in the MB with atherosclerosis group (*P* < 0.05), and there was no statistical difference in other indicators (*P* > 0.05) (Table 1).

### In-group comparison of FAI and PCAT volumes

**Figure 3:**
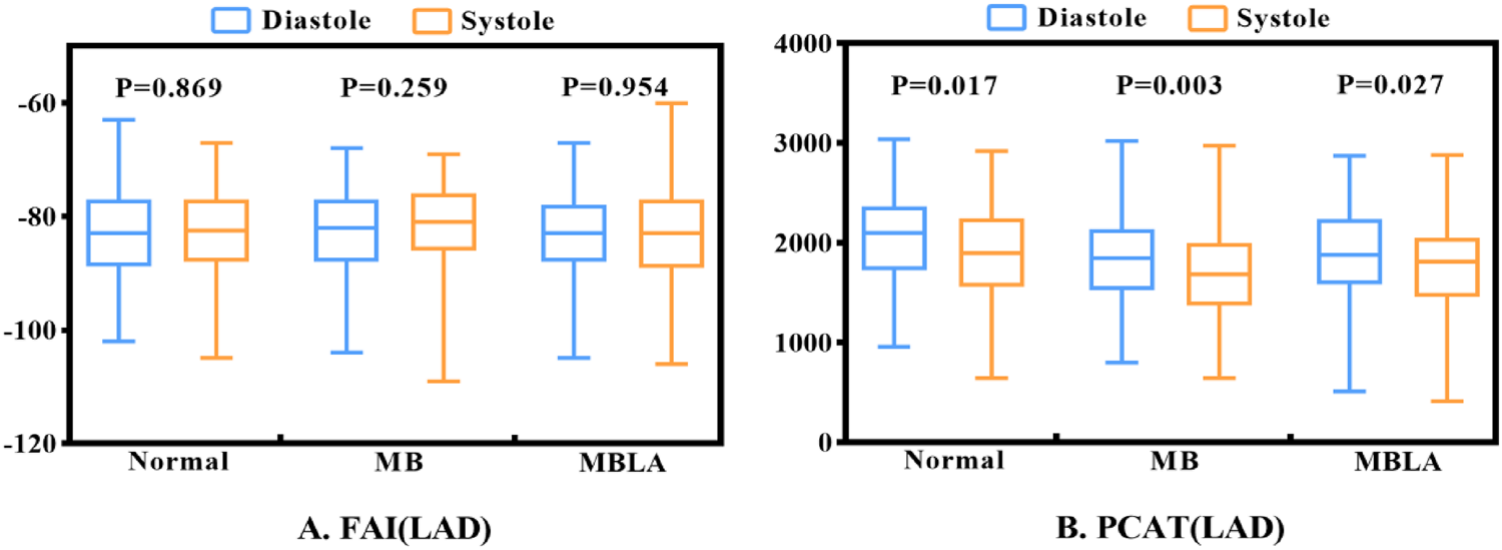
Comparison of the FAI and PCAT values in diastole and in systole in-group. (**A**) FAI was compared in-group. It showed no statistic difference. (**B**) PCAT volume was compared in-group. It was significantly different in diastole and in systole. *FAI* fat attenuation index, *PCAT* pericoronary adipose tissue, *Normal* the control group without MB, *MB* myocardial bridging, *MBLA* myocardial bridging combined with LAD atherosclerosis, *LAD* left anterior descending coronary artery.

The results showed that there was no statistical difference in FAI in systole and in diastole (*P* > 0.05) (Table 1; Fig. 3A). The volumes of PCAT in diastole was greater than that in systole (*P* < 0.05) (Table 1; Fig. 3B).

### Comparison of FAI and the PCAT volumes among the MB group, the MB with atherosclerosis group, and the control group

**Figure 4:**
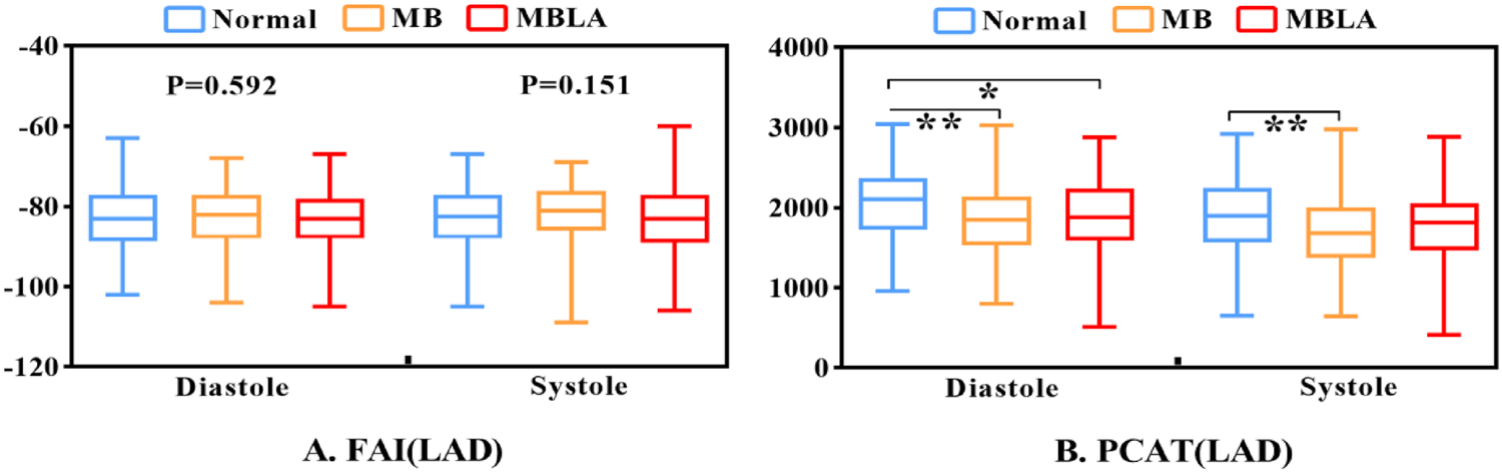
Comparison of the FAI and PCAT values in the diastolic and systolic phase. (**A**) Comparison of the FAI values in the diastolic and systolic phase. (**B**) Comparison of the PCAT volumes in the diastolic and systolic phase. *Normal* the control group without MB, *MB* myocardial bridging, *MBLA* myocardial bridging combined with LAD atherosclerosis, *FAI* fat attenuation index, *PCAT* pericoronary adipose tissue, *LAD* left anterior descending coronary artery; *P < 0.05; **P < 0.01.

The results showed that there was no significant difference in FAI among the three groups in systole and in diastole (*P* > 0.05) (Table 1; Fig. 4A).

The results showed that in the diastole, the PCAT volume of normal group was higher than that of myocardial bridge group and myocardial bridge with atherosclerosis group (*P* < 0.05), but there was no statistical difference between myocardial bridge group and myocardial bridge with atherosclerosis group. In the systolic period, the PCAT volume in the normal group was higher than that in the myocardial bridge group (*P* < 0.05), but there was no difference between the normal group and the myocardial bridge with atherosclerosis group (Table 1; Fig. 4B).

### Analysis of factors influencing FAI in the MB group

Patients in the MB group were divided into the normal FAI group and the abnormal FAI group. There was no statistically significant difference in clinical characteristics and myocardial bridge anatomic characteristics between the two groups (*P* > 0.05; Table 2).

**Table 2:** Comparison of clinical characteristics and anatomical features of MB between patients with normal and abnormal FAI (≥ − 70.1) in the MB group.

Variables	FAI < − 70.1 (n = 145)	FAI ≥ − 70.1 (n = 10)	*P*
Patients			
Age (y)	51.7 ± 10.5	49.1 ± 10.7	0.440
Male (n, %)	77 (53.1)	5 (50)	1.000
BMI	24.6 ± 3.3	25.5 ± 4.6	0.876
Risk factors			
Hypertension (n, %)	32 (22.1)	4 (40.0)	0.362
Hyperlipidemia (n, %)	14 (9.7)	2 (20.0)	0.275
Diabetes (n, %)	14 (9.7)	1 (10.0)	1.000
Smoking (n, %)	24 (16.6)	3 (30.0)	0.380
Symptoms			
Angina pectoris (n, %)	12 (8.3)	1 (10.0)	0.595
Atypical angina (n, %)	9 (6.2)	1 (10.0)	0.497
Chest tightness and pain (n, %)	13 (9.0)	1 (10.0)	1.000
None (n, %)	111 (76.6)	7 (70.0)	0.703
MB			
Length (mm)	24.1 ± 11.4	29.9 ± 10.6	0.080
Depth (mm)	2.0 ± 1.3	2.4 ± 1.4	0.244
Location (mm)	37.6 ± 12.1	31.2 ± 6.8	0.111
Systolic compression index	0.09 ± 0.13	0.11 ± 0.09	0.798
MBMI	53.3 ± 48.0	78.6 ± 63.7	0.098
MB stenosis rate in diastole	0.31 ± 0.13	0.36 ± 0.17	0.170
MB stenosis rate in systole	0.32 ± 0.18	0.35 ± 0.16	0.585

*MB* myocardial bridging, *FAI* fat attenuation index, *BMI* body mass index, *MBMI* MB muscle index.

### Analysis of factors influencing FAI in the MB with atherosclerosis group

**Figure 5:**
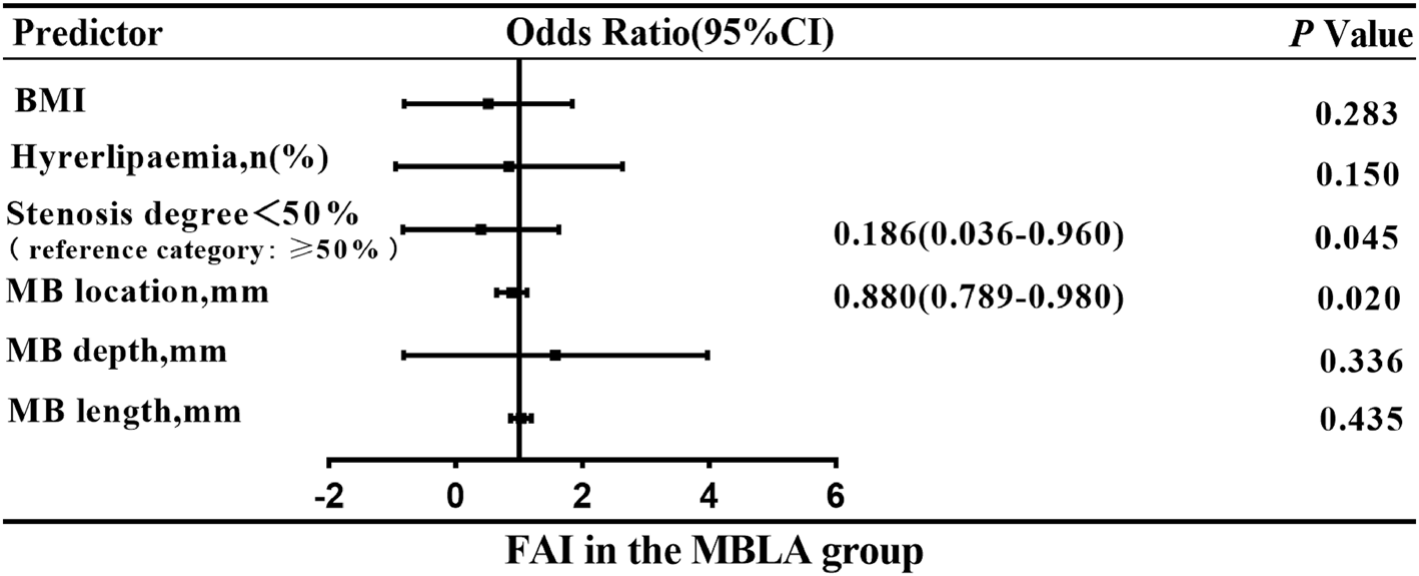
Multivariant logistic regression analysis was performed for prediction of abnormal FAI values in the MB with atherosclerosis group. *FAI* fat attenuation index, *MB* myocardial bridging, *CI* confidence interval.

Patients in the MB with atherosclerosis group were divided into the normal FAI group and the abnormal FAI group. There were statistically significant differences in BMI, length, depth, location and MBMI of myocardial bridge between the two groups (Table 3). The above variables and variables of clinical significance or reported significance were included in the multivariate binary logistic regression analysis. The results showed that LAD stenosis degree < 50% (OR = 0.135, 95% CI 0.027–0.668; *P* = 0.014) and MB position (OR = 0.880, 95% CI 0.789–0.980; *P* = 0.020) were protective factors for abnormal FAI (Fig. 5; Table 4).

**Table 3:** Comparison of clinical characteristics and anatomical features of MB between patients with normal and abnormal FAI (≥ − 70.1) in the MB with atherosclerosis group.

Variables	FAI < 70.1 (n = 134)	FAI ≥ − 70.1 (n = 11)	*P*
Patients			
Age (y)	53.7 ± 9.7	52.5 ± 9.6	0.779
Male (n, %)	77 (57.5)	6 (54.5)	1.000
BMI	25.5 ± 3.0	23.6 ± 3.9	0.037*
Risk factors			
Hypertension (n, %)	40 (29.9)	3 (27.3)	1.000
Hyperlipidemia (n, %)	22 (16.4)	4 (36.4)	0.110
Diabetes (n, %)	16 (11.9)	2 (18.2)	0.628
Smoking (n, %)	15 (11.2)	0 (0.0)	0.605
Symptoms			
Angina pectoris (n, %)	16 (11.9)	2 (18.2)	0.628
Atypical angina (n, %)	8 (6.0)	0 (0.0)	1.000
Chest tightness and pain (n, %)	19 (14.2)	0 (0.0)	0.360
None (n, %)	91 (67.9)	9 (81.8)	0.503
MB			
Length (mm)	22.8 ± 10.6	30.9 ± 10.4	0.006*
Depth (mm)	1.6 ± 0.97	2.6 ± 1.4	0.004*
Location (mm)	40.9 ± 28.4	25.3 ± 9.8	0.000*
Systolic compression index	0.10 ± 0.09	0.04 ± 0.14	0.152
MBMI	40.3 ± 37.5	82.3 ± 58.9	0.000*
MB stenosis rate in diastole	0.31 ± 0.12	0.35 ± 0.16	0.235
MB stenosis rate in systole	0.32 ± 0.13	0.32 ± 0.22	0.307
Plaques			
Calcification (n, %)	64 (37.9)	4 (26.7)	0.466
Non-calcification (n, %)	88 (52.1)	9 (60)	0.339
Mixed (n, %)	17 (10.1)	2 (13.3)	0.638
Coronary artery stenosis			
< 50% (n, %)	89 (66.4)	4 (36.4)	–
≥ 50% (n, %)	45 (33.6)	7 (63.6)	0.056

*MB* myocardial bridging, *MBLA* myocardial bridging and LAD atherosclerosis, *FAI* fat attenuation index; *BMI* body mass index, *MBMI* MB muscle index. *P < 0.05.

**Table 4:** Univariant and multivariant logistic regression analyses of FAI in the MB group and the MB with atherosclerosis group.

Variables	Univariate analysis		Multivariable analysis	
	OR (95% CI)	*P*	OR (95% CI)	*P*
MB: FAI				
MB length (mm)	1.040 (0.989–1.094)	0.125	1.025 (0.951–1.103)	0.520
MB location (mm)	0.946 (0.884–1.012)	0.107	0.956 (0.889–1.029)	0.232
MB muscle index	1.009 (0.998–1.020)	0.125	1.000 (0.983–1.017)	0.991
MB stenosis rate in diastole	16.281 (0.116–2289.273)	0.269	2.553 (0.010–668.239)	0.741
MB with atherosclerosis: FAI				
BMI	0.828 (0.661–1.037)	0.101	0.876 (0.689–1.115)	0.283
Hyperlipidemia (%)	0.344 (0.093–1.275)	0.110	0.303 (0.060–1.540)	0.150
Coronary artery stenotic degree (%)	0.289 (0.080–1.039)	0.057	0.186 (0.036–0.960)	0.045*
MB location (mm)	0.882 (0.815–0.954)	0.002*	0.880 (0.789–0.980)	0.020*
MB depth (mm)	1.923 (1.213–3.049)	0.005*	1.373 (0.720–2.619)	0.336
MB length (mm)	1.060 (1.009–1.113)	0.022*	1.026 (0.962–1.095)	0.435

*FAI* fat attenuation index, *MB* myocardial bridging, *MBLA* myocardial bridging combined with LAD atherosclerosis, *BMI* body mass index. *P < 0.05.

### Predictive value of MB location

**Figure 6:**
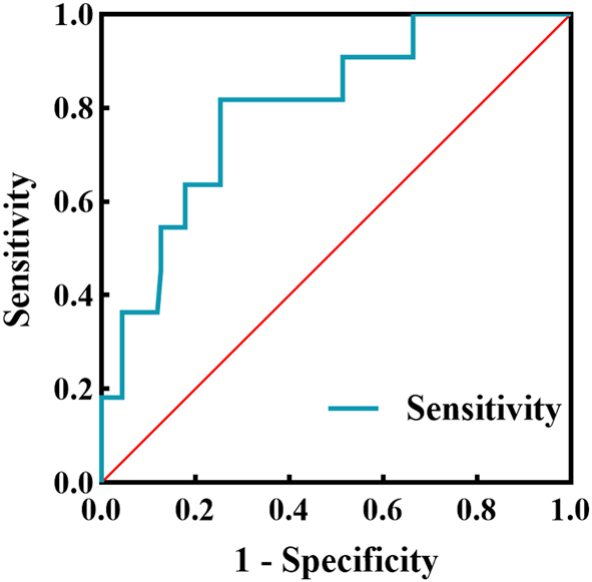
ROC curve analysis of myocardial bridge location in predicting abnormal FAI. The AUC was 0.798 (95% CI 0.669–0.927; *P* = 0.001); Sensitivity: 81.1%, Specificity: 74.6%. The best cut-off value: 29.85. *AUC* area under the curve, *ROC* receiver operating characteristic, *FAI* fat attenuation index.

The ROC curve analysis showed that a distance (from the LAD opening to the proximal point of the MB) of 29.85 mm had the highest predictive value for abnormal FAI [area under the curve (AUC), 0.798], with a sensitivity of 81.1% and a specificity of 74.6% (Fig. 6). MB closer to the LAD opening will incur a relatively higher risk.

## Discussion

PCAT is not only the structural support tissue of blood vessels, but also the endocrine organ with active metabolism, which can produce various pro-inflammatory and anti-inflammatory factors^13^. PCAT is considered to be the key factor to maintain the steady state of cardiovascular physiological function and lead to cardiovascular disease, and can respond to coronary artery inflammation and change the shape and secretion characteristics through the “from inside to outside” signal pathway. These changes can be identified by CTA non-invasive imaging^14^. By calculating FAI, we can non-invasive measure coronary artery inflammation, capture changes in fat attenuation around blood vessels, and predict cardiovascular risk of patients^14^.

Myocardial bridge was first proposed by Dr. Henrick Reyman in 1732^15^. At that time, it was considered as a silent anatomical variation. Most people had no obvious symptoms, but in patients with symptoms, it could lead to serious cardiovascular events^16^, such as serious adverse coronary artery events, myocardial infarction, myocardial ischemia and sudden cardiac death, and the incidence was gradually increasing^17–19^.

This study found that male patients with myocardial bridge had a higher incidence rate and smoking rate, which was consistent with Aydar and Podolec’s research^20,21^. The higher incidence of MB among smokers may be related to the increasing risk of coronary artery spasm by smoking^21^.

Although there have been studies on PCAT volume and FAI value, there is no comparison between systolic and diastolic measurements of those parameters. Our study found that there was no significant difference in FAI values between systolic and diastolic phases measurements, but there was difference in PCAT volumes. The reason for this may be that FAI is the average CT attenuation of PCAT after correction and weighting^22^, which quantifies coronary artery inflammation through the difference of PCAT attenuation gradient^8^, and adjusts some factors affecting PCAT. Therefore, the measurement of FAI value is not affected by the cardiac cycle phase, and may be more suitable than PCAT volume for the evaluation of pericoronary fatty lesions. Oikonomou et al.^8^ showed that FAI was more accurate in predicting cardiovascular diseases and had higher prediction ability. In the diastole, the PCAT volume of normal group was higher than that of myocardial bridge group and myocardial bridge with atherosclerosis group. We speculate that measuring PCAT volume during diastole may be more conducive to detecting changes in pericoronary adipose tissue (PCAT) associated with myocardial bridge or atherosclerotic plaques.

This study found that the clinical symptoms, risk factors and myocardial bridge anatomy were not the influencing factors of FAI abnormalities in patients with myocardial bridge. It shows that myocardial bridge has no effect on the FAI value of pericoronal fat. However, the abnormality of FAI in patients with both myocardial bridge and coronary atherosclerosis is related to the location of myocardial bridge and the degree of vascular stenosis. The closer the location of myocardial bridge to the opening of the LAD, and the greater the degree of stenosis, the more likely it will lead to abnormal FAI values. In other words, pericoronary steatoinflammation increases at the proximal end of the myocardial bridge only in the presence of large atherosclerotic plaques. It is speculated that the closer the myocardial bridge is to the LAD orifice, the greater the intimal thickening at the proximal end^23^, which causes changes in the hemodynamics of the proximal vessel, thus leading to the formation of atherosclerotic plaque^24^, increased inflammation and changes in FAI values at the proximal end of the myocardial bridge.

Vasa vasorum (VV) in the proximal and distal segments of myocardial bridge increased^4^. The increase of VV formation is considered as an inflammatory change of adventitia^4^. The closer the myocardial bridge is to the port of the left coronary artery, the greater the intimal thickening at the proximal end^23^. Generally, coronary atherosclerosis does not occur at the myocardial bridge segment, and atherosclerotic plaque often occurs at the proximal end of the myocardial bridge^24,25^. Local hemodynamic disorder caused by muscle contraction of the myocardial bridge may play a role in plaque formation^24^.

Our research has certain limitations. First of all, this study was a single-center study with a limited number of cases. Second, this study only focused on myocardial bridges located in LAD. Although this is the most common location of myocardial bridges, our data may not be applicable to myocardial bridges in other arteries. Third, the FAI value measured in this study is only the average density and cannot fully reflect the structural characteristics of the fat around the myocardial bridge. In addition, artificial intelligence technology combined with radiomics is expected to provide accurate evaluation of FAI in the future^26,27^.

## Conclusion

Myocardial bridge may affect the volume of pericoronal fat, but it does not affect the pericoronal fat attenuation index. When myocardial bridge patients with LAD atherosclerosis, the degree of coronary artery stenosis < 50% and the location of myocardial bridge away from the LAD opening are protective factors of FAI value.

## Data Availability

The data that support the findings of this study are available on request from the corresponding author.

## Abbreviations

CCTA: Coronary computed tomography angiography
LAD: Left anterior descending coronary artery
MB: Myocardial bridging
FAI: Fat attenuation index
PCAT: Epicardial adipose tissue
OR: Odds ratio
AUC: Area under the curve
ROC: Receiver operating characteristic
